# Is L2 Learners’ Metaphorical Competence Essentially Cognitive, Linguistic, or Personal?—A Meta-Analysis

**DOI:** 10.3390/jintelligence13090117

**Published:** 2025-09-11

**Authors:** Zhaojuan Chen, Lu Guan, Xiaoyong Zhou

**Affiliations:** 1 College of Foreign Studies, Nanjing Agricultural University, Nanjing 210095, China; miachen@njau.edu.cn; 2 School of Foreign Languages, East China Normal University, Shanghai 200241, China; guan_ecnuer@163.com

**Keywords:** metaphorical competence, second language acquisition, meta-analysis, linguistic factors, cognitive factors, personal factors

## Abstract

Metaphorical competence—the capacity to comprehend and produce metaphors in a second language (L2)—is essential for nuanced, accurate, and contextually appropriate English usage. Synthesizing 40 independent studies (N = 15,786), this meta-analysis quantified the relative contributions of cognitive, linguistic, and personal factors to L2 metaphorical competence. Effect sizes were derived from correlation coefficients and aggregated under random-effects models to account for between-study heterogeneity. Linguistic factors emerged as the dominant predictor (r = 0.421, 95% CI [0.34, 0.50]), primarily driven by vocabulary breadth/depth and reading proficiency. Cognitive factors exerted a moderate influence (r = 0.232, 95% CI [0.17, 0.30]), whereas personal variables such as gender yielded only a small effect (r = 0.216, 95% CI [0.15, 0.28]). Moderator analyses further revealed that L1 conceptual knowledge constitutes the strongest single predictor of L2 metaphor skills and highlighted distinct associations between receptive and productive metaphor abilities with linguistic versus cognitive aptitudes. The findings collectively point to lexico-semantic and literacy development as the main levers for boosting L2 metaphorical competence, with cognitive aptitudes and personal factors acting as secondary, yet important, modulators. Insight from this meta-analysis offers a robust foundation for evidence-based decisions in curriculum design, materials selection, and targeted pedagogical interventions.

## 1. Introduction

The acquisition of a second language requires developing a wide range of competencies. From a linguistic competence perspective (Chomsky 1965), learners aim to master skills for understanding and producing diverse sentences. Communicative competence (Hymes 1972) extends this by emphasizing socially appropriate language use, encompassing grammatical, sociolinguistic, discursive, and strategic skills (Savignon 1985). Among these, metaphorical competence is a critical yet underexplored area in second language acquisition research (Castellano-Risco and Piquer-Píriz 2020; O’Reilly and Marsden 2021).

The concept of metaphorical competence emerged in the late 1970s when Gardner and Winner (1978) defined it as the ability to detect, interpret, and create novel figurative mappings. Low (1988) emphasized its importance for second language (L2) learners, describing it as metaphor-related skills native speakers master, essential for competent language use. Danesi (1986, 1992) argued that control of figurative language is critical for true L2 proficiency, introducing “conceptual fluency” to describe the underlying mental ability. Synthesizing prior work, Littlemore (2001) and Littlemore and Low (2006a, 2006b) identified four measurable components—originality of production, fluency of interpretation, ability to find meaning, and speed of interpretation—integrating these into communicative competence’s linguistic, sociolinguistic, discourse, and strategic dimensions. They posited that metaphorical competence is as vital as communicative competence for language proficiency. Azuma (2004) distinguished receptive competence (interpreting metaphors in listening or reading) from productive competence (generating creative metaphors in speaking or writing). This study adopts Azuma’s framework for its clear operational distinction.

Cognitive linguistics theories (Lakoff and Johnson 1980) underscore the ubiquity of metaphors, highlighting the need for L2 learners to develop metaphor comprehension and production skills to enhance proficiency, cross-cultural communication, academic writing, and literary appreciation. Early metaphor research viewed them as linguistic phenomena, but Lakoff and Johnson’s *Metaphors We Live* By (1980) shifted the focus to cognitive linguistics. Danesi (1995) further emphasized conceptual fluency as the ability to align formal structures with underlying conceptual structures in the target language, noting that reliance on L1 thinking often leads to inaccurate or inappropriate L2 speech. This indicates the critical role of metaphorical competence in effective L2 learning.

Given the critical role of metaphorical competence in second language acquisition, this study aims to provide a comprehensive understanding of the factors influencing metaphorical competence in L2 learners.

To clarify the scope of the present synthesis, three broad predictor domains are distinguished:

- Cognitive factors encompass general mental abilities (e.g., fluid intelligence, verbal intelligence) and preferred thinking styles (e.g., holistic vs. analytic, field-dependent vs. field-independent).
- Linguistic factors subsume L2 proficiency indicators (vocabulary breadth/depth, reading comprehension, writing ability, overall proficiency) and L1 metaphorical competence.
- Personal factors include biologically or socially anchored individual-difference variables (gender, personality traits such as introversion/extroversion). Motivation, affect, or identity—although acknowledged as potentially relevant—were not included because (a) there are no empirical research in the metaphor literature or (b) their conceptual overlap with cognitive factors (e.g., self-regulation) would inflate heterogeneity.

The fragmented and sometimes contradictory findings suggest that narrative reviews alone cannot resolve competing theoretical claims. For instance, J. Johnson’s (1991) conclusion that cognitive ability outweighs language proficiency has been challenged by studies demonstrating strong lexical-semantic effects (O’Reilly and Marsden 2021). Similarly, conflicting evidence on gender effects may stem from uncontrolled proficiency covariates. A meta-analytic synthesis is therefore warranted to quantify effect sizes and to test moderator hypotheses (e.g., whether the predictive power of L1 conceptual knowledge attenuates at higher L2 proficiency levels).

By synthesizing evidence from numerous studies, this research aims to quantify the contributions of cognitive, linguistic, and personal factors to metaphorical competence. The goal is to provide insights for curriculum design and teaching practices, enabling educators to develop effective strategies to enhance L2 learners’ metaphorical competence and overall language proficiency.

## 2. Literature Review: Variables That May Impact L2 Learners’ Metaphorical Competence

Metaphorical competence is crucial for second language (L2) learners, yet its influencing factors remain debated. Cognitive factors, such as thinking styles and intelligence, have been linked to metaphorical competence, with some studies suggesting a stronger influence than general language proficiency. Linguistic factors, including L2 proficiency, L1 metaphorical competence, and skills like vocabulary and reading comprehension, are also significant predictors. Personal factors, such as gender and personality, have been explored but show inconsistent effects. Conflicting findings across these domains and the limited scope of individual studies highlight the need for a meta-analysis to quantify the relative effect sizes of cognitive, linguistic, and personal factors.

### 2.1. Cognitive Variables

Research presents conflicting evidence regarding cognitive influences. While (J. Johnson 1991; J. M. Johnson 1996; Johnson and Rosano 1993) asserts cognitive ability outweighs language proficiency in metaphor comprehension—with bilingual children’s performance differences attributed primarily (94%) to cognitive factors—this contrasts sharply with later findings. Littlemore (2001) and Wang and Hao (2013) demonstrate that cognitive style (holistic vs. analytic) significantly impacts interpretation speed and conceptual sensitivity, while Chen et al. (2014) show field-dependent learners benefit more from conceptual metaphor instruction. Li’s (2012) evidence of cognitive-linguistic interdependence further complicates this picture.

### 2.2. Linguistic Variables

The linguistic evidence is robust but methodologically fragmented. Multiple studies confirm L2 proficiency correlates with metaphorical competence (Aleshtar and Dowlatabadi 2014; Rezaei and Jafarpour 2013; Littlemore et al. 2014), with vocabulary (O’Reilly and Marsden 2021; Deng and Xiao 2012; Han 2020) and reading (Zhao et al. 2014) showing particularly strong relationships. Yet, operationalizations vary widely: while Dabbagh and Hashemi (2023) emphasize cultural conceptualization transfer, Zibin (2016) identifies specific metaphor types causing difficulty (e.g., Type 3 with similar expressions but divergent concepts). Crucially, Arif and Abdullah (2017) and Littlemore (2010) demonstrate L1 metaphorical competence transfers to L2, but this effect diminishes with proficiency. This fragmentation obscures which linguistic components are most consequential—precisely why our analysis examines effect sizes across vocabulary, reading, writing, and L1 transfer to establish hierarchy.

### 2.3. Personal Variables

Personal factors remain underexplored with inconsistent methodologies. Hashemian (2018) reports personality types (perceiving vs. judging) significantly influence competence, while Ifantidou and Hatzidaki (2019) suggest non-linguistic elements (emotions, mental imagery) contribute to metaphor processing. However, gender studies show contradictory results: Fattahi and Nushi (2021) identify significant effects where Galantomos (2019) finds none. This inconsistency stems from inadequate sample sizes and failure to control for proficiency covariates. The lack of replicated findings in this domain necessitates our meta-analytic approach to determine whether personal factors warrant instructional consideration or represent negligible influences.

## 3. Research Objectives

While prior research has identified cognitive, linguistic, and personal variables as potential influences on L2 metaphorical competence, the relative magnitude and consistency of these effects remain unclear due to methodological fragmentation and contradictory findings (e.g., J. Johnson 1991 vs. Littlemore 2001; Fattahi and Nushi 2021 vs. Galantomos 2019). To resolve these discrepancies and quantify the precise contributions of each factor category, this meta-analysis addresses the following questions:

(1) What are the comparative effect sizes of cognitive, linguistic, and personal factors on L2 metaphorical competence?
(2) To what extent do these factors collectively and differentially determine L2 learners’ metaphorical competence across receptive and productive domains?
(3) What potential moderators (e.g., L1 conceptual knowledge, proficiency level, measurement tools) explain the observed heterogeneity across studies?

By synthesizing empirical evidence from 15,786 learners, this study aims to establish a hierarchy of predictive factors (cognitive, linguistic, personal) through effect size quantification, identify key moderators (e.g., L1 conceptual knowledge, proficiency level) explaining variance across studies, and provide empirically grounded insights for instructional prioritization.

## 4. Methodology

The significant heterogeneity in methodological approaches, variable definitions, and reported effect sizes across studies investigating factors influencing L2 learners’ metaphorical competence necessitates the application of meta-analysis. This quantitative synthesis approach was selected as the most appropriate method for this research due to its unique capacity to systematically aggregate and statistically integrate findings from diverse empirical studies (Borenstein et al. 2009). Such methodological rigor aligns precisely with the core objectives of this study: to resolve conflicting findings in the literature (e.g., J. Johnson 1991 vs. Littlemore 2001; Fattahi and Nushi 2021 vs. Galantomos 2019) through objective quantification; to establish the relative magnitude and consistency of effects across different predictor variables (cognitive, linguistic, personal); and to identify potential moderators (e.g., proficiency level, L1 background, measurement tools) that might explain the observed variability in outcomes reported by scholars like Chen et al. (2014), O’Reilly and Marsden (2021), and Dabbagh and Hashemi (2023). Crucially, meta-analysis directly addresses the limitations of narrative reviews by providing statistically robust answers to questions central to this investigation, particularly the necessity of metaphorical competence development and its integrative relationship with other linguistic and cognitive skills, thereby achieving the research objectives effectively.

### 4.1. Article Identification Process

We began our study with literature identification. The identification process was conducted independently by the two researchers. Whenever there were any divergences between the two authors, they would discuss them until a consensus was reached. We used Summon, a locally customized academic search service the authors’ university provides, as our primary search engine. Databases included in this service are Web of Science, EBSCO, SpringerLink, ScienceDirect, Scopus, Wiley Online Library, etc. We used Google Scholar as our secondary search engine. The search terms we used are listed in Table 1.

The primary search on Summon yielded a total of 3610 results. To locate studies that are not appropriately indexed in electronic databases (Hopewell et al. 2005), an additional manual search was conducted using Google Scholar, which yielded an additional 22 results.

### 4.2. Eligibility Criteria

The eligibility criteria for the present review were the following:

- Topics of research: studies aimed at exploring metaphors or metaphorical competencies in L2 learning and teaching.
- Type of research: empirical research examining correlational effects between any variable and the metaphorical competence of L2 learners.
- Date of publication: Studies published since 1980 were considered. This starting year was chosen because it marked the publication of “*Metaphors We Live By*”, signaling a cognitive shift in metaphor conceptualization.
- Language of publication: Studies in English and Chinese were included to capture research published in the predominant language for academia as well as literature from the authors’ native language, given these two languages’ wide global usage and large speaker bases.
- Forms of publication: original research paper, review paper, degree thesis, book chapter, and book.
- Research methods: studies presenting sufficient statistical information about the data (e.g., sample size; correlation *r*, *t*, or *p*-value) to calculate an average effect size.
- Exclusion criteria were as follows:
- Studies that were not written in English or Chinese.
- Studies for which no full-text publication was available. Example: A study on developmental connections among L1 conceptual transfer competence, metaphoric competence, and English proficiency (Shi 2012).
- Studies focusing on L1 language learning rather than L2 language learning; for example: metaphor processing in middle childhood and at the transition to early adolescence: the role of chronological age, mental age, and verbal intelligence (Deckert et al. 2019).
- Studies that focused on metaphors/conceptual metaphors without directly referring to language learning; for example: *Metaphorical Expressions and Culture: An Indirect Link* (Deignan 2003).
- Studies that partly address metaphor in second language but primarily deal with figurative language in general. Example: *Figurative Thinking and Foreign Language Learning* (Littlemore and Low 2006a).
- Studies that are either theoretical or speculative. Example: *Metaphor and Second Language Learning: The State of the Field* (Hoang 2014).
- Studies reporting results from regression modeling from which zero-order correlation cannot be sufficiently derived. Example: *Fluency or Similarities? Cognitive Abilities that Contribute to Creative Metaphor Generation* (Kasirer and Mashal 2018).

After removing duplicate literature, the authors independently evaluated the remaining articles using predefined inclusion and exclusion criteria. Articles accepted by both authors were included, while those deemed unacceptable were excluded. Disagreements were resolved through discussion until consensus was reached. Ultimately, 81 articles were deemed relevant to our study. Additionally, we used the snowballing method to identify further literature, recognizing that database and manual searches might miss pertinent sources (Greenhalgh and Peacock 2005). We cross-referenced the refined article list to identify citations absent from initial and supplementary searches, adding two more relevant articles to our study.

### 4.3. Coding Process

To examine the characteristics of primary studies and identify potential moderators for inconsistent findings, we implemented a systematic process to code study features for each article in our meta-analysis. The coding, conducted by the authors, occurred in three phases.

In the first phase, “trial coding”, a team of applied linguistics scholars and methodologists with meta-analysis experience developed a tentative coding plan. The coding table included all study features potentially relevant to later analysis. Two coders independently applied this table to five primary studies, discussing issues and revising the table as needed. In phase two, the coders independently coded all primary studies using the revised table, resolving issues through ongoing discussions. In phase three, the coding results were cross-checked and validated, then integrated into the final coding table. Discrepancies prompted further discussions and review of relevant primary studies for clarification.

Out of the 81 primary studies, 3 were found to have inconsistencies between the coders’ results, yielding a coding consistency rate of 96.38% [(83−3)/83 = 96.38%], indicating high consistency across multiple study features.

Of the 81 studies assessed, 40 provided sufficient statistical data for effect size computation. One study was excluded due to incomplete data after an unanswered request for additional information. The search process is illustrated in Appendix A using a PRISMA Flow Diagram (Page et al. 2021). Many studies reported findings from multiple indicators or independent samples, yielding multiple effect sizes per study. In total, 146 effect sizes were extracted from the 40 studies (N = 15,786). To maintain the meta-analysis assumption of effect size independence, studies with multiple outcomes were treated as distinct estimates (see the Appendix B for detailed coding). This approach may slightly underestimate the standard error of point estimates (Borenstein et al. 2009). However, prioritizing information retention outweighed concerns about this minor statistical violation.

### 4.4. Publication Bias

To address potential publication bias, this study used multiple literature identification methods and sought unpublished studies (e.g., doctoral dissertations) when feasible. Two standard techniques were applied to test for publication bias.

**Figure 1:**
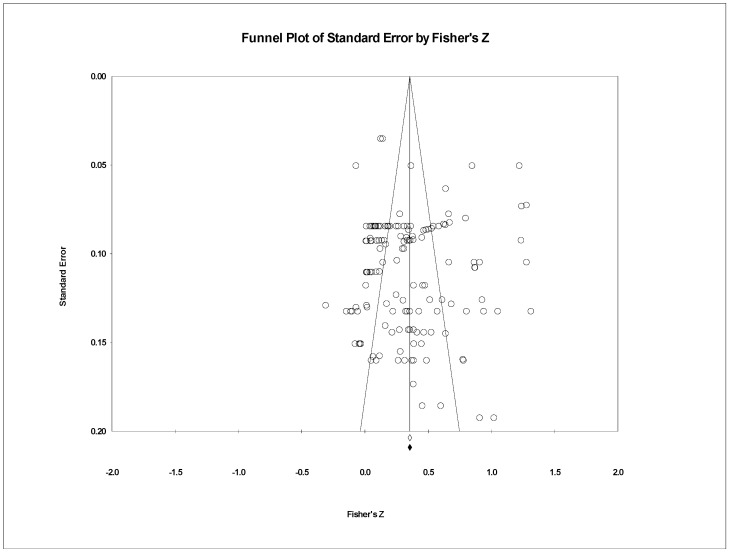
Funnel plot of standard error by Fisher’s Z.

First, effect sizes were visually examined using a funnel plot (Figure 1). The plot showed effect sizes more evenly distributed at the lower part of the “inverted funnel”, with fewer studies at the top, suggesting possible publication bias, as smaller studies with insignificant results may be underrepresented.

**Figure 2:**
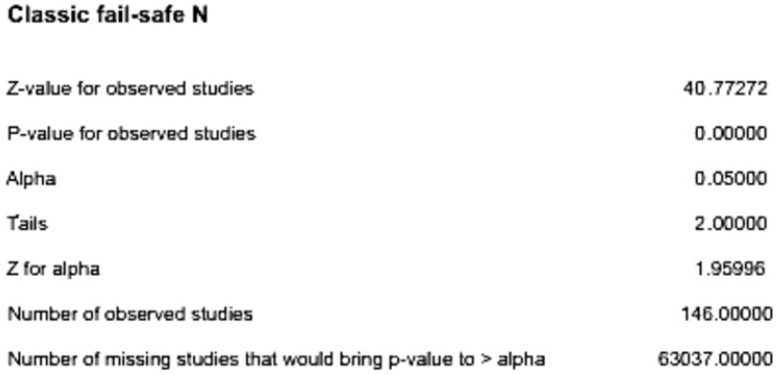
Classic fail-safe N.

Moreover, quantitative assessment using the failsafe factor method (Nfs) yielded an Nfs value of 63,037, far exceeding the threshold of 5k + 10 (where k = 145, the number of effect sizes). This indicates that 63,037 additional studies would be needed to shift the combined 2-tailed *p*-value above 0.05 (Figure 2), suggesting minimal impact from publication bias (Figure 2).

### 4.5. Computation of Effect Size

CMA 3.0 (Comprehensive Meta Analysis 3.0) software was used to perform meta-analysis statistics in this study. To integrate the results of each study, the correlation coefficient *r* was used as the effect size. For meta-analysis, correlation coefficient r values were transformed into Fisher Z-values, taking into account the statistical errors caused by sample characteristics, study context, and sample size. Z-tests were used to assess the statistical significance of the weights assigned to each study based on the sample size.

Several papers (Arif and Abdullah 2017, for example) included in this study did not report correlation coefficients but rather reported regression coefficients. We computed r by using the equation r = β × 0. 98 + 0. 05 (β ≥ 0) to calculate the value of r (Peterson and Brown 2005).

### 4.6. Heterogeneity Test

Meta-analysis employs various models with distinct assumptions. Fixed-effect models assume all within-study variance stems from measurement error, while random-effects models account for both heterogeneous true effects across studies and within-study measurement errors. To determine the appropriate model for this study, a heterogeneity test was conducted, with the results presented in Table 2. Heterogeneity is typically assessed using the Q-test and I^2^ statistic. The Q-test, based on total variance, assumes effect sizes follow a chi-square distribution, with *p* < 0.05 indicating heterogeneity. The I^2^ statistic quantifies the percentage of total variation due to differences between studies rather than sampling error, with I^2^ = 25% indicating low heterogeneity, I^2^ = 50% moderate heterogeneity, and I^2^ = 75% high heterogeneity (Table 2).

The results of the heterogeneity test in this study showed that the Q value was 241.977 (*p* < 0.001), which was significant. Moreover, the I^2^ value was 74.790, also indicating a high level of heterogeneity. Therefore, a random model was chosen for the present study.

## 5. Results

### 5.1. Overall Effects for All the Variables

Following rigorous assessment of publication bias and heterogeneity (see Section 4), our meta-analysis revealed a significant aggregate effect size of r = 0.344 (95% CI [0.28, 0.41]) across all predictor variables on L2 metaphorical competence (Table 3). Per Cohen’s (1988) benchmarks, this represents a moderate overall influence, indicating that while cognitive, linguistic, and personal factors collectively contribute to metaphorical ability, their combined impact leaves substantial variance unexplained by these domains alone. Crucially, this synthesis suggests that metaphorical competence emerges not from isolated factors but from dynamic interactions between learners’ cognitive architectures, language proficiency, and individual differences.

### 5.2. Summary Effects for the Three Categories of Variables

Our subgroup analyses revealed a clear hierarchy of influences on L2 metaphorical competence (Table 4). Critically, linguistic factors demonstrated the strongest predictive power (r = 0.421, 95% CI [0.34, 0.50]), approaching a large effect size. This robust association—substantially larger than other domains—underscores that metaphor processing depends fundamentally on language-specific resources: vocabulary depth, reading proficiency, and metalinguistic awareness collectively enable learners to decode and produce figurative expressions.

Cognitive factors showed a small-to-moderate relationship (r = 0.232, CI [0.17, 0.30]), suggesting that while abilities like verbal intelligence and holistic thinking facilitate metaphorical reasoning, they play a secondary role to linguistic mastery. This nuance resolves theoretical debates between cognitivist (e.g., J. Johnson 1991) and applied linguistic (e.g., Littlemore 2001) perspectives by quantifying their relative contributions.

Similarly, personal factors like gender exhibited only marginal influence (r = 0.216, CI [0.15, 0.28]), indicating that individual differences in biologically anchored traits minimally impact metaphor acquisition when linguistic and cognitive foundations are accounted for. This hierarchy (Linguistic > Cognitive > Personal) persisted in sensitivity analyses, confirming its robustness across methodological variations.

To examine whether the larger number of linguistic studies (k = 87) disproportionately influenced the overall findings, we conducted a leave-one-domain-out sensitivity analysis. After re-running the meta-analysis three times—each time excluding one of the three dimensions—the rank order of effect sizes remained Linguistic > Cognitive > Personal, and the pooled effect shifted by less than 0.015. Moreover, we present separate meta-analytic means and prediction intervals for each dimension, thereby preventing the larger number of effect sizes in linguistic dimensions from masking uncertainty in the smaller sets.

In order to investigate whether other factors such as metaphorical competence types, cognitive types or intelligence, and language skill types have effects on L2 students’ metaphorical competence, we also conducted moderator analyses within subgroups.

### 5.3. Moderator Variables Within the Cognitive Subgroup

As indicated in Table 5, analysis of cognitive moderators revealed distinct patterns: when measured as intelligence constructs (verbal/fluid intelligence, creativity; k = 20), cognition demonstrated stronger predictive validity for metaphorical competence (r = 0.301) than when defined as stylistic preferences (holistic/analytic thinking; k = 22). Concurrently, the strength of cognitive associations varied substantially across competence types, exhibiting the strongest correlation with general metaphorical competence (r = 0.422, k = 9), moderate effects on receptive skills (r = 0.218, k = 16), and minimal impact on productive abilities (r = 0.139, k = 17). These differential patterns collectively indicate that cognitive factors function as dimensionally contingent predictors whose influence is maximized when (a) operationalized as domain-general reasoning capacities rather than stylistic preferences and (b) measured against broad competence assessments rather than discrete productive tasks (Table 6).

### 5.4. Moderator Variables Within the Linguistic Subgroup

The analysis of linguistic factors revealed that both language skill types and metaphorical competence types significantly moderated effects on L2 metaphorical competence, demonstrating a clear predictive hierarchy. L1 metaphorical competence emerged as the strongest predictor (r = 0.585), indicating cross-linguistic conceptual transfer as the primary mechanism driving metaphor acquisition. General language proficiency (r = 0.520) and writing skills (r = 0.405) similarly showed large effects, confirming that literacy-based metalinguistic awareness substantially scaffolds metaphorical processing, whereas speaking skills exhibited non-significant effects (r = 0.075), suggesting distinct processing demands for oral metaphor production (Table 7). Moreover, linguistic predictors demonstrated differential efficacy across competence types: general competence measurements yielded large effects (r = 0.442) and receptive skills showed near-identical large effects (r = 0.455), while productive abilities generated moderate effects (r = 0.366), revealing a consistent advantage for interpretation over generation tasks (Table 8).

### 5.5. Moderator Variables Within the Personal Subgroup

The analysis of personal factors revealed limited predictive utility, with gender differences (examined in 15 of 17 studies) demonstrating only a small aggregate effect on L2 metaphorical competence (r = 0.194), indicating minimal influence relative to linguistic and cognitive domains. Personality traits (e.g., introversion/extroversion) could not be statistically evaluated due to insufficient primary studies (k = 2), as reflected in Table 9. Further analysis of gender’s differential effects across competence types (Table 10) showed a moderate impact on general metaphorical competence (r = 0.319, k = 4), while revealing consistently small effects for both receptive (r = 0.173, k = 3) and productive (r = 0.118, k = 8) dimensions.

Across 146 effect sizes drawn from 40 studies (N = 15,786), a clear hierarchy of influence emerged: linguistic factors explained the largest share of variance in L2 metaphorical competence (r = 0.421, 95% CI [0.34, 0.50]), approaching a large effect; cognitive factors displayed a moderate-to-small association (r = 0.232, CI [0.17, 0.30]); and personal variables—chiefly gender—exerted only a marginal influence (r = 0.216, CI [0.15, 0.28]). Sensitivity analyses confirmed that this rank ordering (Linguistic > Cognitive > Personal) remained stable when any single domain was omitted. Moderator findings further revealed that (1) L1 metaphorical knowledge and overall L2 proficiency were the strongest linguistic predictors; (2) verbal/fluid intelligence outweighed cognitive-style measures; and (3) receptive metaphor skills were more tightly linked to reading proficiency, whereas productive skills hinged on broader language ability. Taken together, the evidence establishes that metaphorical competence is primarily a linguistic accomplishment, modestly supported by cognitive resources, and minimally affected by personal traits—a pattern that persisted across methodological variations and metaphor-competence types.

## 6. Discussion

The statistical hierarchy reported above—Linguistic (r = 0.421) > Cognitive (r = 0.232) > Personal (r = 0.216)—is more than a ranking of effect sizes. Read through a developmental lens, it suggests that metaphorical competence in a second language is first and foremost a linguistic milestone whose emergence is scaffolded by incremental mastery of lexico-semantic and discourse knowledge, and only secondarily by domain-general cognitive maturation.

### 6.1. Linguistic Factors as Primary Enablers

Across 87 linguistic effect sizes, pooled effects reached r = 0.42, surpassing cognitive and personal predictors. Viewed through a developmental lens, this suggests language-specific resources serve as threshold competencies essential for efficient figurative processing. This aligns with emergentist accounts (e.g., Low 1988), which view metaphor comprehension as probabilistic mapping across lexical-constructional networks, collapsing without sufficient semantic depth.

Vocabulary breadth and depth emerged as the key sub-component (r = 0.378; O’Reilly and Marsden 2021; Deng and Xiao 2012; Han 2020). From a usage-based perspective, each additional lemma expands potential source domains and strengthens collocational probabilities, reducing the processing load for novel figurative uses (Charteris-Black 2002). Reading proficiency complements this: sustained exposure to printed metaphors provides distributional evidence to consolidate polysemous senses and register-specific mappings (Zhao et al. 2014). Thus, vocabulary and reading effects are multiplicative—rich lexical stores enhance gains from reading, and vice versa.

Cross-sectional correlations (Aleshtar and Dowlatabadi 2014; Rezaei and Jafarpour 2013; Littlemore et al. 2014) indicate the linguistic threshold varies by proficiency: at upper-intermediate levels, general proficiency gains yield diminishing metaphor returns, signaling a shift from lexical retrieval to conceptual mapping. Pedagogically, this supports a staged curriculum: intensive vocabulary expansion and strategy-based reading instruction should precede creative metaphor production tasks, supporting calls for explicit lexico-grammatical scaffolding before figurative extension (Lazar 1996; Littlemore and Low 2006b).

### 6.2. Moderate Role of Cognitive Dimensions

Cognitive abilities—intelligence and thinking style—exert a measurable but moderate effect (*r* ≈ 0.23). Re-examined through a developmental lens, this finding reframes cognition not as the primary engine of metaphor acquisition, but as a mediator whose influence is gated by linguistic readiness. Johnson’s early claim that cognitive ability outweighs language proficiency in children’s metaphor comprehension (J. Johnson 1991; J. M. Johnson 1996; Johnson and Rosano 1993) can now be read as reflecting a developmental phase: when linguistic variability among young L1 speakers is small, cognitive differences become the salient differentiator. In the far more heterogeneous linguistic terrain of adult L2 learners, however, the same cognitive resources are recruited through the lexico-grammatical system (cf. Lakoff and Johnson 1980).

Mediational evidence supports this view. Verbal intelligence enhances metaphor aptitude chiefly by expanding lexical access and semantic encoding capacities—skills that overlap heavily with linguistic competencies (Chiappe and Chiappe 2007). Roth et al. (1996) likewise demonstrate that vocabulary size partially mediates the relationship between intelligence and reading comprehension.

Moderator analyses further nuance this picture. Verbal and fluid intelligence generated larger effects than stylistic variables such as holistic versus analytic processing, yet the latter still modulated task performance. Littlemore (2001) found that holistic style accelerated meaning-discovery speed, while Wang and Hao (2013) linked analytic style to more accurate metaphorical comprehension—patterns consistent with a resource-allocation account in which cognitive style reallocates attentional resources within an already available linguistic workspace. Creativity, though under-investigated, appears to amplify metaphor production once linguistic and cognitive floors are secure (Kövecses 2010). Thus, cognitive factors function less as independent engines and more as amplifiers that magnify the reach of existing linguistic resources.

### 6.3. Personal Factors as Socially Mediated Micro-Processes

Across the 17 studies addressing gender or personality, pooled effects hovered around r = 0.20. Viewed purely as a main-effect variable, then, gender appears to exert minimal influence on L2 metaphorical competence. Yet, developmental and sociolinguistic theory caution against equating statistical non-significance with theoretical irrelevance. Sunderland (2010) argues that gender operates through differential access to metaphor-rich registers—academic, narrative, or affective—rather than through a direct causal pathway. In this light, the negligible aggregate effect documented here may reflect an over-reliance on decontextualized measures that strip away the very socialization histories in which gendered language practices are forged.

A socio-cognitive reframing therefore invites future work to model gender as a moderator rather than a predictor. Similarly, personality factors such as openness or tolerance of ambiguity could interact with instructional style: learners high in openness may profit more from open-ended metaphor generation tasks, whereas more analytic learners benefit from explicit mapping instructions.

In sum, the present meta-analysis confirms that personal traits do not drive metaphor acquisition in the way linguistic and cognitive resources do. Nevertheless, a developmental perspective suggests they may still channel how linguistic and cognitive resources are mobilized across tasks, contexts, and developmental stages. Longitudinal designs that embed learners in their socio-educational ecologies are needed to surface these contingent pathways.

### 6.4. Moderator Variable Insights

The moderator analyses yielded two key insights with direct pedagogical relevance for L2 metaphor instruction. First, the robust predictive power of L1 conceptual competence (r = 0.585, *p* < 0.001) underscores its foundational role in L2 metaphorical development. This large effect size—the strongest among linguistic subcomponents—confirms that learners’ existing metaphoric frameworks in their native language significantly scaffold L2 figurative processing (Danesi 2016; Dabbagh and Atai 2022). Rather than treating L1 as interference, instructors should strategically leverage cross-linguistic comparisons to accelerate conceptual fluency. For example, contrastive analysis tasks (e.g., comparing how time metaphors operate as “money” in English versus “water” in Mandarin) can explicitly highlight conceptual alignments and divergences, thereby facilitating positive transfer while mitigating negative transfer (Zibin 2016; Arif and Abdullah 2017). Such activities align with the meta-analytic evidence that learners with heightened metalinguistic awareness of L1–L2 metaphor mappings demonstrate superior L2 interpretive accuracy.

Second, the differential relationships between receptive (r = 0.455) and productive (r = 0.361) metaphor skills and their underlying aptitudes necessitate tailored pedagogical approaches. Receptive competence correlated more strongly with reading proficiency (r = 0.232) and holistic cognitive style, suggesting instruction should prioritize scaffolded exposure to metaphors in authentic texts (e.g., annotating conventional metaphors in academic readings) paired with schema-building tasks that activate conceptual mappings (Boers 2000). Conversely, productive competence relied more heavily on vocabulary depth (r = 0.378) and verbal intelligence, indicating that guided generation exercises—such as rewriting literal sentences figuratively or creating novel extensions of conceptual metaphors (e.g., “time is a thief” → “deadlines rob me of creativity”)—are essential to bridge lexical knowledge to creative output (Kövecses 2010; O’Reilly and Marsden 2021). This evidence-based differentiation resolves the “one-size-fits-all” approach to metaphor teaching, urging curricula to address receptive and productive skills through distinct cognitive-linguistic pathways.

## 7. Conclusions

This meta-analysis synthesized findings from 40 studies to quantify the relative influence of cognitive, linguistic, and personal factors on L2 learners’ metaphorical competence. The results provide valuable insights into the knowledge and skills integral to metaphor interpretation and production in a second language.

### 7.1. Pedagogical Implications

This meta-analysis demonstrates that linguistic factors, notably vocabulary knowledge and reading proficiency, are the strongest predictors of L2 metaphorical competence, explaining over 40% of variance (r = 0.421). These findings align with theories emphasizing the critical role of semantic and discourse skills in processing figurative language. Cognitive factors, such as verbal intelligence and thinking style, have a moderate influence (r = 0.232), indicating that metaphor aptitude depends more on linguistic proficiency than general cognitive abilities. Personal factors, like gender, contribute minimally (r = 0.216). L1 conceptual knowledge strongly predicts L2 metaphor skills (r = 0.585), with receptive and productive metaphor abilities showing distinct relationships to linguistic and cognitive aptitudes.

To develop L2 metaphorical competence effectively, educators should integrate metaphor instruction into broader literacy initiatives. This leverages the finding that linguistic factors account for the largest proportion of variance (r = 0.421), confirming metaphor aptitude emerges from general language proficiency. For instance, teachers might embed metaphor analysis in reading workshops or incorporate metaphor journals into vocabulary units documenting conceptual mappings like “IDEAS AS PLANTS” (r = 0.378), reinforcing the interdependence of vocabulary, reading, and metaphorical processing.

In light of the findings of this meta-analysis, some suggestions for the teaching of L2 metaphorical competence can be made.

To develop L2 metaphorical competence effectively, educators should **integrate metaphor instruction into broader literacy initiatives**. This leverages the meta-analytic finding that linguistic factors accounted for the largest proportion of variance (r = 0.421), confirming metaphor aptitude emerges from general language proficiency. For instance, teachers might embed metaphor analysis in reading workshops or incorporate metaphor journals into vocabulary units documenting conceptual mappings like “IDEAS AS PLANTS”, reinforcing the interdependence of vocabulary, reading, and metaphorical processing.

**Explicit instruction in vocabulary depth and reading strategies** is crucial for developing metaphor skills. Given the strong vocabulary–metaphor correlation (r = 0.378), activities like polysemy mapping, idiom decoding, and collocation analysis should be prioritized. Reading tasks should scaffold metaphor detection through literal–figurative annotation or inferring conceptual mappings.

Beyond direct vocabulary and reading instruction, learners benefit from **a parallel track that cultivates meta-linguistic awareness** of how metaphors are built, used, and varied. Meta-linguistic reflection acts as a cognitive lever: once learners can articulate the conceptual mapping underlying a figurative expression, they become better able to transfer that mapping to new lexical items and new contexts.

Instruction should **strategically leverage learners’ L1 conceptual knowledge** through cross-linguistic comparison. The significant effect size of L1 metaphorical competence (r = 0.585) supports Danesi’s (2016) conceptual fluency hypothesis, suggesting that comparing L1–L2 metaphor systems accelerates acquisition. Practical applications include contrastive analysis of emotion encodings, spatial metaphors, or cultural logic in idioms, etc. This cultivates the “meta-cultural competence” Dabbagh and Hashemi (2023) identified as critical for navigating conceptual divergences.

Supplementing general language courses with **tailored metaphor modules** addresses underdeveloped dimensions. These might include deconstructing metaphor typologies, analyzing register variation in figurative language, or examining socio-cultural influences, etc. Pairing explicit metaphor instruction with incidental exposure would yield the strongest gains in metaphorical competence.

Finally, **cultivating creative problem solving** through metaphor innovation tasks taps into cognitive aptitudes. While cognitive factors showed moderate overall influence (effect size = 0.232), verbal intelligence emerged as a key predictor. Activities like conceptual blending or genre-shifting align with Kövecses’ (2010) observation that novel metaphor production engages fluid intelligence. Such tasks motivate learners while developing the cognitive flexibility needed for advanced L2 proficiency.

### 7.2. Limitations

However, several limitations should be acknowledged.

First, the 40 studies employed either receptive, productive, or hybrid metaphor tasks; this instrument heterogeneity (I^2^ = 74.8%) partly explains the effect-size dispersion. Vocabulary predicted receptive items (r = 0.455) more strongly than creative production (r = 0.139), whereas fluid intelligence showed the reverse. Future work should employ latent-variable modelling or cross-task generalizability studies to disentangle construct variance from method variance.

Moreover, the present synthesis was constrained by a pronounced imbalance in the empirical record: 87 studies examined linguistic predictors of metaphorical competence, compared with 42 for cognitive factors and only 17 for personal factors. While random-effects modelling and sensitivity analyses confirm that no single domain unduly drives the pooled estimates, the larger corpus on linguistic variables necessarily yields narrower confidence intervals and higher statistical power. This asymmetry enhances the apparent robustness of linguistic effects but does not in itself confer superior validity; the relative sparsity of data on cognitive and personal dimensions leaves their true effect sizes more uncertain and potentially underestimated. Consequently, the field would benefit from deliberately designed studies that target under-represented cognitive abilities (e.g., fluid intelligence, cognitive style) and personal attributes (e.g., personality traits, gender intersections) so that future meta-analyses can re-evaluate the hierarchy of influences on L2 metaphorical competence with more evenly distributed evidence.

Thirdly, our systematic search located only one eligible book chapter. Consequently, the meta-analytic estimate for this source type is based on minimal data, potentially under-representing book-based evidence. Future studies should revisit emerging handbooks and edited volumes.

Finally, the literature reviewed was restricted to English and Chinese studies. More cross-linguistic data could strengthen the validity and generalizability of the findings. We therefore caution readers against extrapolating the current hierarchy to typologically distant languages. Studies conducted in languages with richer inflectional morphology or evidential systems resources is an urgent next step for validating or refining the present research.

In summary, this meta-analysis highlights the integral role of linguistic skills in L2 learners’ metaphor competence. Educational initiatives should emphasize building vocabulary, reading, and discourse proficiency to enhance metaphor interpretation and production. While cognitive and personal factors contribute modestly, linguistic capabilities appear most decisive in determining one’s figurative aptitude. Further research can extend these findings by using more targeted measures of metaphor types and language knowledge.

## Figures and Tables

**Table 1:** Search term combinations.

Primary Search Term	Secondary Search Term
*metaphorical competence*	*language learning*
*metaphorical intelligence*	*language teaching*
*Metaphorical thinking*	*second language*
*conceptual metaphor*	*foreign language*
*conceptual fluency*	*L2 Language*
*Metaphor*	

*Note*. We combined the primary search terms with the secondary search terms during the systematic search period, and a total of 30 searches were performed.

**Table 2:** Results of the heterogeneity test.

Number of Studies	Q-Value	df (Q)	*p*-Value	I-Squared
146	241.977	61	0.000	74.790

**Table 3:** Overall effects for all variables.

Models	Effect Size and 95% Confidence Interval						Test of Null (2-Tail)	
Model	Number Studies	Point Estimate	Standard Error	Variance	Lower Limit	Upper Limit	Z	*p*
Fixed	146	0.345	0.008	0.000	0.328	0.361	43.344	0.000
Random effects	146	0.344	0.028	0.001	0.288	0.399	12.167	0.000

**Table 4:** Subgroup effect sizes.

Groups	Effect Size and 95% Confidence Interval						Test of Null (2-Tail)	
Group	Number Studies	Point Estimate	Standard Error	Variance	Lower Limit	Upper Limit	Z Value	*p* Value
Cognitive	42	0.232	0.033	0.001	0.168	0.296	7.072	0.000
Linguistic	87	0.421	0.041	0.002	0.340	0.502	10.188	0.000
Personal	17	0.216	0.035	0.001	0.147	0.284	6.198	0.000

**Table 5:** Cognitive measurements as moderator variables within the cognitive subgroup.

Groups	Effect Size and 95% Confidence Interval						Test of Null (2-Tail)	
Group	Number Studies	Point Estimate	Standard Error	Variance	Lower Limit	Upper Limit	Z Value	*p* Value
Cognitive intelligence	20	0.301	0.058	0.003	0.188	0.414	5.233	0.000
Cognitive style	22	0.171	0.033	0.001	0.105	0.236	5.138	0.000

**Table 6:** Metaphorical competence types as moderator variables within the cognitive subgroup.

Groups	Effect Size and 95% Confidence Interval						Test of Null (2-Tail)	
Group	Number Studies	Point Estimate	Standard Error	Variance	Lower Limit	Upper Limit	Z Value	*p* Value
General MC	9	0.422	0.069	0.005	0.287	0.556	6.134	0.000
Productive MC	17	0.139	0.034	0.001	0.073	0.205	4.125	0.000
Receptive MC	16	0.218	0.059	0.003	0.103	0.333	3.705	0.000

**Table 7:** Language skill types as moderator variables within the linguistic subgroup.

Groups	Effect Size and 95% Confidence Interval						Test of Null (2-Tail)	
Group	Number Studies	Point Estimate	Standard Error	Variance	Lower Limit	Upper Limit	Z Value	*p* Value
General proficiency	26	0.520	0.063	0.004	0.396	0.644	8.222	0.000
L1 metaphorical competence	12	0.585	0.126	0.016	0.338	0.833	4.629	0.000
Listening	13	0.319	0.097	0.009	0.129	0.509	3.285	0.001
Reading	8	0.232	0.069	0.005	0.096	0.367	3.351	0.001
Speaking	3	0.075	0.049	0.002	−0.020	0.171	1.547	0.122
Vocabulary	17	0.378	0.045	0.002	0.290	0.466	8.442	0.000
Writing	8	0.405	0.180	0.032	0.053	0.757	2.256	0.02

**Table 8:** Metaphorical competence types as moderator variables within the linguistic subgroup.

Groups	Effect Size and 95% Confidence Interval						Test of Null (2-Tail)	
Group	Number Studies	Point Estimate	Standard Error	Variance	Lower Limit	Upper Limit	Z Value	*p* Value
General MC	30	0.442	0.073	0.005	0.298	0.586	6.031	0.000
Productive MC	27	0.361	0.077	0.006	0.211	0.512	4.697	0.000
Receptive MC	30	0.455	0.067	0.004	0.323	0.586	6.790	0.000

**Table 9:** Different measurements as moderator variables within the personal subgroup.

Groups	Effect Size and 95% Confidence Interval						Test of Null (2-Tail)	
Group	Number Studies	Point Estimate	Standard Error	Variance	Lower Limit	Upper Limit	Z Value	*p* Value
Gender	15	0.194	0.034	0.001	0.127	0.256	5.675	0.000
Personal	2	0.219	0.094	0.009	0.240	0.545	4.570	0.000

**Table 10:** Metaphorical competence types as moderator variables within the gender subgroup.

Groups	Effect Size and 95% Confidence Interval						Test of Null (2-Tail)	
Group	Number Studies	Point Estimate	Standard Error	Variance	Lower Limit	Upper Limit	Z Value	*p* Value
General MC	4	0.319	0.055	0.003	0.211	0.427	5.792	0.000
Productive MC	3	0.118	0.063	0.004	−0.05	0.241	1.877	0.061
Receptive MC	8	0.173	0.046	0.002	0.083	0.263	3.766	0.000

**Figure A1:**
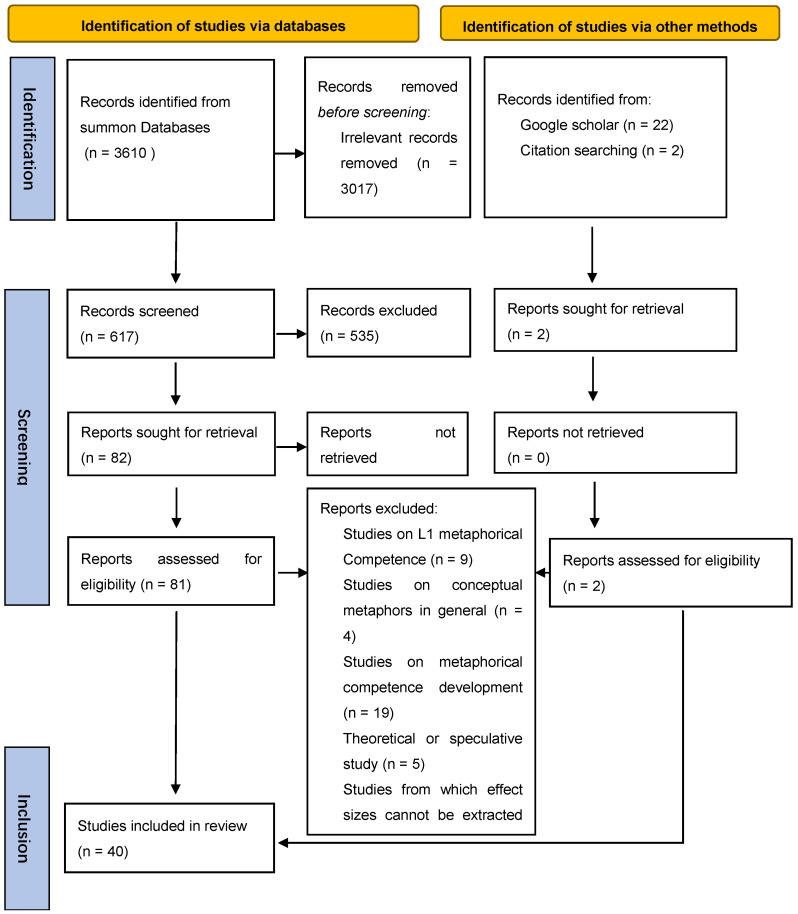
Article identification process.

## Data Availability

Data is contained within Appendix B.

## Appendix B. Data Entry Form

**Number**	**Study Name**	**Outcome**	**Data Format**	**A Mean**	**A Std-Dev**	**A Sample** **Size**	**B Mean**	**B Std-Dev**	**B Sample** **Size**	**Effect** **Direction**	**Correlation**	**Std Err**	**Fisher’s Z**	**Std Err**
1	Chen 20141	cognitive	Independent groups (means, SDs)	80.07	14.05	26	87.75	10.32	14	3	0.273243	0.143552	0.280365	0.155135
2	Chen 20142	cognitive	Independent groups (means, SDs)	87	12.25	26	84.67	23.94	14	3	6.46 × 10^−2^	0.15729	6.47 × 10^−2^	0.157949
3	Chen 20143	cognitive	Independent groups (means, SDs)	85.07	15.02	26	88.38	9.97	14	3	0.116099	0.155456	0.116625	0.15758
4	Chen 20144	cognitive	Independent groups (means, SDs)	97.75	14.09	26	79.67	9.24	14	3	0.56352	9.90 × 10^−2^	0.637976	0.145019
5	Hashemian 20181	cognitive	Independent groups (means, SDs)	17.03	1.45	42	17.37	0.91	48	3	0.140833	0.102805	0.141775	0.104885
6	Hashemian 20182	cognitive	Independent groups (means, SDs)	16.97	1.25	54	17.58	1.03	36	3	0.247974	9.74 × 10^−2^	0.253253	0.103776
7	Kenett 2018	cognitive	Independent groups (means, SDs)	0.86	0.06	32	0.83	0.06	32	3	0.242536	0.115904	0.247466	0.123148
8	Littemore 20105	cognitive	Independent groups (means, SDs)	2.83	0.34	46	2.82	0.32	36	3	1.50 × 10^−2^	0.110401	1.50 × 10^−2^	0.110425
9	Littemore 20106	cognitive	Independent groups (means, SDs)	1.37	0.51	46	1.35	0.42	36	3	2.10 × 10^−2^	0.110371	2.10 × 10^−2^	0.110419
10	Littemore 20107	cognitive	Independent groups (means, SDs)	3.03	0.43	46	3.01	0.52	36	3	2.10 × 10^−2^	0.11037	2.10 × 10^−2^	0.110419
11	Littemore 20108	cognitive	Independent groups (means, SDs)	6.23	2.18	46	6.79	2.64	36	3	0.115399	0.108598	0.115916	0.110063
12	Yuan 20200	cognitive	Independent groups (means, SDs)	5.03	3.79	58	5.8	4.45	58	3	9.27 × 10^−2^	0.091851	9.30 × 10^−2^	9.26 × 10^−2^
13	Yuan 202011	cognitive	Independent groups (means, SDs)	7.55	5.62	58	7.65	5.63	58	3	8.89 × 10^−3^	9.28 × 10^−2^	8.89 × 10^−3^	9.28 × 10^−2^
14	Yuan 202012	cognitive	Independent groups (means, SDs)	14.72	10.65	58	15.75	11.62	58	3	4.62 × 10^−2^	9.26 × 10^−2^	4.62 × 10^−2^	9.28 × 10^−2^
15	Yuan 20205	cognitive	Independent groups (means, SDs)	3.87	20.6	58	4.27	2.07	58	3	1.37 × 10^−2^	0.092826	1.37 × 10^−2^	9.28 × 10^−2^
16	Yuan 20206	cognitive	Independent groups (means, SDs)	10.37	5.57	58	11.7	6.19	58	3	0.112225	9.14 × 10^−2^	0.1127	9.26 × 10^−2^
17	Yuan 20207	cognitive	Independent groups (means, SDs)	12.95	6.9	58	14.55	7.31	58	3	0.111844	9.14 × 10^−2^	0.112314	9.26 × 10^−2^
18	Yuan 20208	cognitive	Independent groups (means, SDs)	27.18	14.25	58	30.52	15.35	58	3	0.11205	9.14 × 10^−2^	0.112522	9.26 × 10^−2^
19	Yuan 20209	cognitive	Independent groups (means, SDs)	2.13	1.48	58	2.3	1.73	58	3	5.27 × 10^−2^	0.092525	5.28 × 10^−2^	9.28 × 10^−2^
20	Cai 20188	cognitive	Corr, N								0.423	7.46 × 10^−2^	0.45134	9.09 × 10^−2^
21	Hashemian 2012	cognitive	Corr, N								0.277	8.32 × 10^−2^	0.28443	9.02 × 10^−2^
22	Hashemian 2019	cognitive	Corr, N								0.36	7.85 × 10^−2^	0.376886	9.02 × 10^−2^
23	Li 20121	cognitive	Corr, N								0.545	8.86 × 10^−2^	0.611241	0.125988
24	Li 20122	cognitive	Corr, N								0.473	9.78 × 10^−2^	0.513928	0.125988
25	Li 20123	cognitive	Corr, N								0.729	5.90 × 10^−2^	0.92659	0.125988
26	Liu 20184	cognitive	Corr, N								0.322	8.29 × 10^−2^	0.333877	9.25 × 10^−2^
27	Liu 20185	cognitive	Corr, N								0.152	9.03 × 10^−2^	0.153187	9.25 × 10^−2^
28	Liu 20186	cognitive	Corr, N								0.133	9.08 × 10^−2^	0.133793	9.25 × 10^−2^
29	Liu 20187	cognitive	Corr, N								0.344	8.15 × 10^−2^	0.358622	9.25 × 10^−2^
30	Wang 20131	cognitive	Corr, N								0.42	0.124162	0.447692	0.150756
31	Wang 20132	cognitive	Corr, N								−0.077	0.149862	#######	0.150756
32	Wang 20133	cognitive	Corr, N								−0.045	0.15045	#######	0.150756
33	Wang 20134	cognitive	Corr, N								−0.031	0.150611	#######	0.150756
34	Wang 20135	cognitive	Corr, N								0.369	0.130229	0.387265	0.150756
35	Wang 20136	cognitive	Corr, N								−0.038	0.150538	#######	0.150756
36	Wang 20162	cognitive	Corr, N								0.27	7.20 × 10^−2^	0.276864	7.76 × 10^−2^
37	Wei 20091	cognitive	Corr, N								0.342	0.126148	0.356356	0.142857
38	Wei 20092	cognitive	Corr, N								0.331	0.127206	0.343951	0.142857
39	Wei 20093	cognitive	Corr, N								0.267	0.132673	0.273631	0.142857
40	Wei 20094	cognitive	Corr, N								0.367	0.123616	0.384952	0.142857
41	Wei 20121	cognitive	Corr, N								0.701	5.48 × 10^−2^	0.869264	0.107833
42	Xu 20121	cognitive	Corr, N								0.124	0.034682	0.124641	3.52 × 10^−2^
43	Fattahi 20212	linguistic	Independent groups (means, SDs)	63.93	14.35	27	84.09	12.7	23	3	0.593695	8.31 × 10^−2^	0.683353	0.128356
44	Galantomos 20182	linguistic	Independent groups (means, SDs)	0.32	0.09	15	0.45	0.06	16	3	0.649889	9.21 × 10^−2^	0.775106	0.159518
45	Shi 20121	linguistic	Independent groups (means, SDs)	49	15.49	60	66	9.47	62	3	0.553476	5.78 × 10^−2^	0.623379	8.33 × 10^−2^
46	Shi 20122	linguistic	Independent groups (means, SDs)	32	17.62	60	72	34.84	62	3	0.584735	5.42 × 10^−2^	0.669628	8.24 × 10^−2^
47	Shi 20123	linguistic	Independent groups (means, SDs)	81	27.78	60	138	36.07	62	3	0.662016	4.49 × 10^−2^	0.796395	0.080003
48	Suner 20181	linguistic	Independent groups (means, SDs)	4.233	1.294	60	0.817	0.813	60	3	0.845072	2.09 × 10^−2^	1.238656	7.32 × 10^−2^
49	Suner 20182	linguistic	Independent groups (means, SDs)	4.65	1.505	60	2.6	1.509	60	3	0.5624	5.73 × 10^−2^	0.636336	8.38 × 10^−2^
50	Suner 20183	linguistic	Independent groups (means, SDs)	3.064	1.187	60	4.35	20.9	60	3	4.34 × 10^−2^	0.091072	4.34 × 10^−2^	9.12 × 10^−2^
51	Yuan 20201	linguistic	Independent groups (means, SDs)	4.07	2.06	60	2.22	1.6	60	3	0.448299	6.92 × 10^−2^	0.48257	8.66 × 10^−2^
52	Yuan 20202	linguistic	Independent groups (means, SDs)	11.03	5.87	60	5.42	4.12	60	3	0.484026	6.57 × 10^−2^	0.528229	8.58 × 10^−2^
53	Yuan 20203	linguistic	Independent groups (means, SDs)	13.75	7.1	60	7.6	5.58	60	3	0.433879	7.05 × 10^−2^	0.464665	8.69 × 10^−2^
54	Yuan 20204	linguistic	Independent groups (means, SDs)	28.85	14.78	60	15.23	11.06	60	3	0.462547	6.78 × 10^−2^	0.500546	8.63 × 10^−2^
55	Aleshtar 20141	linguistic	Corr, N								0.77	7.83 × 10^−2^	1.020328	0.19245
56	Aleshtar 20142	linguistic	Corr, N								0.72	9.27 × 10^−2^	0.907645	0.19245
57	Aleshtar 20143	linguistic	Corr, N								0.782	0.051455	1.050498	0.132453
58	Arif 2017	linguistic	Corr, N								0.602	4.04 × 10^−2^	0.696278	6.34 × 10^−2^
59	Cai 20189	linguistic	Corr, N								0.318	0.081716	0.329421	9.09 × 10^−2^
60	Deng 20122	linguistic	Corr, N								0.481	0.110943	0.524284	0.144338
61	Deng 20123	linguistic	Corr, N								0.212	0.13785	0.215265	0.144338
62	Deng 20124	linguistic	Corr, N								0.435	0.117025	0.466047	0.144338
63	Deng 20125	linguistic	Corr, N								0.39	0.122384	0.4118	0.144338
64	Dong 20141	linguistic	Corr, N								−0.056	0.132038	-0.05606	0.132453
65	Dong 20142	linguistic	Corr, N								−0.111	0.130821	-0.11146	0.132453
66	Dong 20143	linguistic	Corr, N								−0.145	0.129668	-0.14603	0.132453
67	Dong 20144	linguistic	Corr, N								0.865	3.33 × 10^−2^	1.312871	0.132453
68	Dong 20145	linguistic	Corr, N								0.666	7.37 × 10^−2^	0.80352	0.132453
69	Dong 20146	linguistic	Corr, N								0.735	6.09 × 10^−2^	0.939516	0.132453
70	Han 20200	linguistic	Corr, N								0.09	0.158831	9.02 × 10^−2^	0.160128
71	Han 20203	linguistic	Corr, N								0.355	0.139948	0.371153	0.160128
72	Han 20204	linguistic	Corr, N								0.453	0.127268	0.488468	0.160128
73	Han 20205	linguistic	Corr, N								0.305	0.145232	0.315023	0.160128
74	Han 20206	linguistic	Corr, N								0.367	0.138561	0.384952	0.160128
75	Han 20207	linguistic	Corr, N								0.05	0.159728	5.00 × 10^−2^	0.160128
76	Han 20208	linguistic	Corr, N								0.258	0.149469	0.263965	0.160128
77	Han 20209	linguistic	Corr, N								0.652	9.21 × 10^−2^	0.77877	0.160128
78	Hoang 20151	linguistic	Corr, N								0.35	4.43 × 10^−2^	0.365444	5.04 × 10^−2^
79	Hoang 20152	linguistic	Corr, N								0.84	1.49 × 10^−2^	1.221174	5.04 × 10^−2^
80	Hoang 20153	linguistic	Corr, N								−0.07	5.02 × 10^−2^	#######	5.04 × 10^−2^
81	Hoang 20154	linguistic	Corr, N								0.69	2.64 × 10^−2^	0.847956	5.04 × 10^−2^
82	Kou 20171	linguistic	Corr, N								0.537	0.132147	0.59993	0.185695
83	Kou 20172	linguistic	Corr, N								0.425	0.152154	0.453779	0.185695
84	Leng 20141	linguistic	Corr, N								0.517	9.70 × 10^−2^	0.572237	0.132453
85	Leng 20142	linguistic	Corr, N								0.322	0.11872	0.333877	0.132453
86	Leng 20143	linguistic	Corr, N								0.403	0.110942	0.427225	0.132453
87	Leng 20144	linguistic	Corr, N								0.218	0.126159	0.221555	0.132453
88	O’Reilly 20170	linguistic	Corr, N								0.088	8.39 × 10^−2^	8.82 × 10^−2^	8.45 × 10^−2^
89	O’Reilly 20171	linguistic	Corr, N								0.323	7.57 × 10^−2^	0.334993	8.45 × 10^−2^
90	O’Reilly 201711	linguistic	Corr, N								0.108	8.35 × 10^−2^	0.108423	8.45 × 10^−2^
91	O’Reilly 201712	linguistic	Corr, N								0.184	8.17 × 10^−2^	0.18612	8.45 × 10^−2^
92	O’Reilly 201713	linguistic	Corr, N								0.041	8.44 × 10^−2^	4.10 × 10^−2^	8.45 × 10^−2^
93	O’Reilly 201714	linguistic	Corr, N								0.011	8.45 × 10^−2^	1.10 × 10^−2^	8.45 × 10^−2^
94	O’Reilly 201715	linguistic	Corr, N								0.083	8.39 × 10^−2^	8.32 × 10^−2^	8.45 × 10^−2^
95	O’Reilly 201716	linguistic	Corr, N								0.196	8.13 × 10^−2^	0.198569	8.45 × 10^−2^
96	O’Reilly 201717	linguistic	Corr, N								0.259	7.88 × 10^−2^	0.265036	8.45 × 10^−2^
97	O’Reilly 201718	linguistic	Corr, N								0.071	0.084089	7.11 × 10^−2^	8.45 × 10^−2^
98	O’Reilly 201719	linguistic	Corr, N								0.08	8.40 × 10^−2^	8.02 × 10^−2^	8.45 × 10^−2^
99	O’Reilly 20172	linguistic	Corr, N								0.348	7.43 × 10^−2^	0.363166	8.45 × 10^−2^
100	O’Reilly 201720	linguistic	Corr, N								0.055	8.43 × 10^−2^	5.51 × 10^−2^	8.45 × 10^−2^
101	O’Reilly 201721	linguistic	Corr, N								0.179	0.081807	0.180949	8.45 × 10^−2^
102	O’Reilly 20173	linguistic	Corr, N								0.159	8.24 × 10^−2^	0.160361	8.45 × 10^−2^
103	O’Reilly 20174	linguistic	Corr, N								0.526	6.11 × 10^−2^	0.584599	8.45 × 10^−2^
104	O’Reilly 20175	linguistic	Corr, N								0.243	7.95 × 10^−2^	0.24796	8.45 × 10^−2^
105	O’Reilly 20176	linguistic	Corr, N								0.494	6.39 × 10^−2^	0.541338	8.45 × 10^−2^
106	O’Reilly 20177	linguistic	Corr, N								0.299	7.70 × 10^−2^	0.308421	8.45 × 10^−2^
107	O’Reilly 20178	linguistic	Corr, N								0.122	8.33 × 10^−2^	0.122611	8.45 × 10^−2^
108	O’Reilly 20179	linguistic	Corr, N								0.107	0.083548	0.107411	8.45 × 10^−2^
109	Rezaei 20126	linguistic	Corr, N								0.844	2.66 × 10^−2^	1.234918	9.25 × 10^−2^
110	Shi 20124	linguistic	Corr, N								0.312	0.11956	0.32276	0.132453
111	Shi 20125	linguistic	Corr, N								0.341	0.117051	0.355224	0.132453
112	Shi 20126	linguistic	Corr, N								0.018	0.130147	0.018002	0.130189
113	Shi 20127	linguistic	Corr, N								−0.069	0.129569	#######	0.130189
114	Tang 20195	linguistic	Corr, N								0.288	8.91 × 10^−2^	0.296384	9.71 × 10^−2^
115	Tang 20196	linguistic	Corr, N								0.3	0.088387	0.30952	9.71 × 10^−2^
116	Tang 20197	linguistic	Corr, N								0.3	0.088387	0.30952	9.71 × 10^−2^
117	Tang 20198	linguistic	Corr, N								0.12	9.57 × 10^−2^	0.120581	9.71 × 10^−2^
118	Wang 20161	linguistic	Corr, N								0.58	5.15 × 10^−2^	0.662463	7.76 × 10^−2^
119	Wang 20201	linguistic	Corr, N								0.856	2.80 × 10^−2^	1.278183	0.104828
120	Wang 20202	linguistic	Corr, N								0.72	5.05 × 10^−2^	0.907645	0.104828
121	Wang 20203	linguistic	Corr, N								0.698	5.38 × 10^−2^	0.86339	0.104828
122	Wang 20204	linguistic	Corr, N								0.581	6.94 × 10^−2^	0.663971	0.104828
123	Wei 20122	linguistic	Corr, N								0.702	5.47 × 10^−2^	0.871233	0.107833
124	Xiao 2016	linguistic	Corr, N								−0.3	0.11748	-0.30952	0.129099
125	Yuan 2014	linguistic	Corr, N								−0.1	0.131129	-0.10034	0.132453
126	Zhao 20101	linguistic	Corr, N								0.367	0.101978	0.384952	0.117851
127	Zhao 20141	linguistic	Corr, N								0.428	9.63 × 10^−2^	0.457446	0.117851
128	Zhao 20142	linguistic	Corr, N								0.008	0.117844	8.00 × 10^−3^	0.117851
129	Zhao 20143	linguistic	Corr, N								0.442	9.48 × 10^−2^	0.474714	0.117851
130	Fattahi 20211	personal	Independent groups (means, SDs)	70.79	18.45	28	76.27	14.46	22	3	0.159638	0.136936	0.161016	0.140517
131	Galantomos 20181	personal	Independent groups (means, SDs)	0.35	0.087	14	0.42	0.09	17	3	0.36567	0.150298	0.383416	0.173497
132	Hashemian 2012b	personal	Independent groups (means, SDs)	16.43	1.77	51	17.43	1.06	75	3	0.332845	7.70 × 10^−2^	0.346025	8.66 × 10^−2^
133	Littemore 20101	personal	Independent groups (means, SDs)	2.82	0.33	15	2.83	0.33	67	3	1.17 × 10^−2^	0.110413	1.17 × 10^−2^	0.110428
134	Littemore 20102	personal	Independent groups (means, SDs)	1.4	0.4	15	1.35	0.48	67	3	4.14 × 10^−2^	0.110195	4.14 × 10^−2^	0.110384
135	Littemore 20103	personal	Independent groups (means, SDs)	2.94	0.53	15	3.05	0.46	67	3	0.089548	0.109326	8.98 × 10^−2^	0.11021
136	Littemore 20104	personal	Independent groups (means, SDs)	6.2	2.61	15	6.54	2.36	67	3	5.46 × 10^−2^	0.110021	0.054614	0.110349
137	Liu 20141	personal	Independent groups (means, SDs)	64.6	10.1662	30	64.9	11.4119	30	3	1.39 × 10^−2^	0.129068	1.39 × 10^−2^	0.129093
138	Liu 20142	personal	Independent groups (means, SDs)	24	10.5373	30	18	8.9378	30	3	0.293527	0.115407	0.302421	0.126288
139	Liu 20143	personal	Independent groups (means, SDs)	32.7	13.7997	30	27.983	13.4321	30	3	0.17066	0.124423	0.172346	0.128156
140	Tang 20191	personal	Independent groups (means, SDs)	41.13	13.679	23	52.367	12.587	87	3	0.335824	8.22 × 10^−2^	0.349378	9.26 × 10^−2^
141	Tang 20192	personal	Independent groups (means, SDs)	5.957	4.193	23	10.827	5.243	87	3	0.365291	7.98 × 10^−2^	0.382978	9.21 × 10^−2^
142	Tang 20193	personal	Independent groups (means, SDs)	11.391	3.564	23	13.724	2.852	87	3	0.30055	8.48 × 10^−2^	0.310124	9.32 × 10^−2^
143	Tang 20194	personal	Independent groups (means, SDs)	23.782	9.94	23	27.861	9.966	87	3	0.164268	9.21 × 10^−2^	0.16577	9.47 × 10^−2^
144	Aidinlou 20171	personal	Corr, N								0.434	0.117151	0.464814	0.144338
145	Xu 20122	personal	Corr, N								0.14	3.45 × 10^−2^	0.140926	3.52 × 10^−2^
146	Aidinlou 20172	personal	Corr, N								0.381	0.105223	0.401229	0.123091
